# Efficacy of ganglion impar block combined with pudendal nerve pulsed radiofrequency for pudendal neuralgia management—a randomized clinical trial

**DOI:** 10.1186/s13063-024-08152-3

**Published:** 2024-05-13

**Authors:** Jiao Ran, Fan Lu, Le Xu, Yu Du, Li Liu, Tao Qi, Xiaoli Zhou, Yulin Zhang, Dong Liu, Rurong Wang, Xuehan Li

**Affiliations:** 1 Department of Anesthesiology, West China Hospital, Sichuan University, Chengdu, China; 2 Department of Pain Medicine, West China Hospital, Sichuan University, Chengdu, China; 3 Department of Anesthesiology, Sichuan Provincial People’s Hospital, University of Electronic Science and Technology of China, No. 32, West Section 2, 1St Ring Road, Qingyang District, Chengdu City, Sichuan Province China; 4 Sichuan Science City Hospital, No. 64 Mianshan Road, Youxian District, Mianyang City, Sichuan Province China; 5 Department of Pain Management, The Affiliated Hospital of Chengdu University of Traditional Chinese Medicine, No. 39, 12 Qiao Road, Jinniu District, Chengdu, China; 6 No.903 Hospital, the Affiliated Hospital of Chengdu Medical College, No. 9 Huafeng New Village, Middle Section of Taiping Road, Jiangyou City, Mianyang City, China; 7 Department of Pain Management, The Second Clinical College of North Sichuan Medical College, Nanchong Central Hospital (Beijing Anzhen Hospital Nanchong Hospital), No. 97, Renmin South Road, Shunqing District, Nanchong, 637000 Sichuan China; 8 Department of Pain Management, Chengdu Second People’s Hospital, No. 10 Qingyunnan Street, Jinjiang District, Chengdu, China

**Keywords:** Ganglion impar block, Pudendal neuralgia, Pudendal nerve block, Pulsed radiofrequency

## Abstract

**Background:**

Pudendal neuralgia is a chronic and debilitating condition. Its prevalence ranges from 5 to 26%. Currently, therapeutic approaches to treat pudendal neuralgia include patient education, medication management, psychological and physical therapy, and procedural interventions, such as nerve block, trigger point injections, and surgery. Drug therapy has a limited effect on pain relief. A pudendal nerve block may cause a significant decrease in pain scores for a short time; however, its efficacy significantly decreases over time. In contrast, pudendal nerve pulsed radiofrequency can provide pain relief for 3 months, and ganglion impar block has been widely used for treating chronic perineal pain and chronic coccygodynia. This study aimed to determine the efficacy and safety of monotherapy (pudendal nerve pulsed radiofrequency) and combination therapy (pudendal nerve pulsed radiofrequency plus ganglion impar block) in patients with pudendal neuralgia.

**Methods:**

This randomized, controlled clinical trial will include 84 patients with pudendal neuralgia who failed to respond to drug or physical therapy. Patients will be randomly assigned into one of the two groups: mono or combined treatment groups. The primary outcome will be a change in pain intensity measured using the visual analog scale. The secondary outcomes will include a Self-Rating Anxiety Scale score, Self-Rating Depression Scale score, the use of oral analgesics, the Medical Outcomes Study Health Survey Short Form-36 Item score, and the occurrence of adverse effects. The study results will be analyzed using intention-to-treat and per-protocol analyses. Primary and secondary outcomes will be evaluated between the mono and combined treatment groups. Subgroup analyses will be conducted based on the initial ailment, age, and baseline pain intensity. The safety of the treatment will be assessed by monitoring adverse events, which will be compared between the two groups.

**Discussion:**

This study protocol describes a randomized, controlled clinical trial to determine the efficacy and safety of mono and combination therapies in patients with pudendal neuralgia. The study results will provide valuable information on the potential benefits of this combination therapy and contribute to the development of more effective and safer treatments for patients with pudendal neuralgia.

**Trial registration:**

Chinese Clinical Trial Registry (ChiCTR2200061800).

## Background

Pudendal neuralgia (PN), also called Alcock’s syndrome or pudendal nerve entrapment syndrome, is characterized by sensations of burning pain, numbness, or paranesthesia across the pudendal nerve (anywhere between the anus and the clitoris) [1]. PN has been attributed to several factors, including inflammation, compression of the pudendal nerve between the sacrotuberous and sacrospinous ligaments, or compression at the pudendal canal level, which are commonly associated with childbirth, trauma, surgical sequelae, or intense bicycling [2]. This condition is chronic and debilitating. Its prevalence ranges from 5 to 26% [3], but may be significantly higher compared with that described in the literature. Patients may feel embarrassed to seek medical advice, whereas the diagnosis of PN can be challenging for health professionals because of the lack of specialized training in this area [4].

Therapeutic approaches to PN include patient education, medication management, psychological and physical therapy, and consideration of procedural interventions, including nerve block, trigger point injections, and surgery [5, 6]. Tricyclic antidepressants (amitriptyline), serotonin-norepinephrine reuptake inhibitors (duloxetine), and antiepileptics (gabapentin) are used as initial monotherapy for PN [6]. The number of patients required to be treated to achieve 50% pain relief from neuropathic pain ranges from 4 to 10, and the inadequate response of neuropathic pain to drug therapy constitutes a highly unmet need [7]. Surgery is the last resort for patients who have been suffering from PN for longer than a year. The remarkable benefit of surgery is the duration of pain relief, which can range from long-term (commonly 4 years) to permanent [5]; however, surgery is invasive. Therefore, therapeutic nerve blocks must be performed prior to referral to surgery [5, 8].

Pudendal nerve block (PNB) is an essential diagnostic tool and treatment method for PN. PNB can significantly decrease pain scores for a short period of time; however, its effect significantly decreases over time [5, 6, 8]. Labat et al. [2] reported that only 13% of patients experienced pain relief, with up to 50% lasting for 90 days following PNB. Kastler et al. [9] found that 6 months post-PNB, the clinical efficacy was 25.2%. Neuromodulation is a commonly used treatment approach for chronic pelvic pain; however, traditional continuous radiofrequency may damage nerves and may be irreversible. Continuous radiofrequency (CRF) is a widely used method for managing conditions, such as zygomatic joint osteoarthritis, trigeminal neuralgia, and occipital neuralgia. CRF may relieve PN, but may cause bowel, bladder, and sexual dysfunction [10]. Pulsed radiofrequency (PRF) is an alternative approach to avoid irreversible damage, in which the temperature is set in a range of 42–50 °C. PRF is effective for radicular pain from spinal diseases, post-herpetic neuralgia, and occipital neuralgia [11]. Fang et al. [10] showed that pudendal nerve PRF combined with PNB provides longer-lasting pain relief and alleviates depression in patients compared with PNB. Ji et al. [12] found that PN was alleviated after 6 months in 88.9% of the patients who received ultrasound-guided high-voltage long-duration PRF. At long-term follow-up (ranging from 2.3 years to 8.8 years), Krijnen et al. found that 89% of patients who received repeated PRF with a median interval duration of 3 months between two sessions described their condition as “(very) much better” [13]. No difference was observed between PNB and PRF immediately after the procedure, although PRF provided significant improvement in pain for 2 weeks to 3 months [12, 14].

Chronic pain is associated with the sympathetic nervous system, which may be treated with sympathetic nerve blocks. Ganglion impar is the terminal solitary ganglion of the bilateral paravertebral sympathetic chains that transmits pelvic and perineal nociceptive messages to the central nervous system. It primarily innervates the perineum, distal rectum, perianal, distal urethra, scrotum, and distal 1/3 of the vagina. Ganglion impar block (GIB) was first used in 1990 to alleviate pelvic cancer pain, and it is frequently targeted for the treatment of perineal and pelvic pain. Currently, GIB is widely used for treating chronic perineal pain and chronic coccygodynia [15–17]. Thus, ganglion impar, acting as vector of nociceptive messages between the visceral and central nervous systems, represents another therapeutic target in PN.

Ultrasound-guided PRF of the pudendal nerve combined with or without GIB is used to treat PN; however, a randomized controlled trial focused on whether pudendal nerve pulsed radiofrequency combined with ganglion impar block is more effective than pudendal nerve pulsed radiofrequency is lacking. Hence, we hypothesize that combined therapy (pudendal nerve pulsed radiofrequency + ganglion impar block) is more effective compared with monotherapy (pudendal nerve pulsed radiofrequency) for long-term improvement of PN.

## Methods

### Study design

**Fig. 1:**
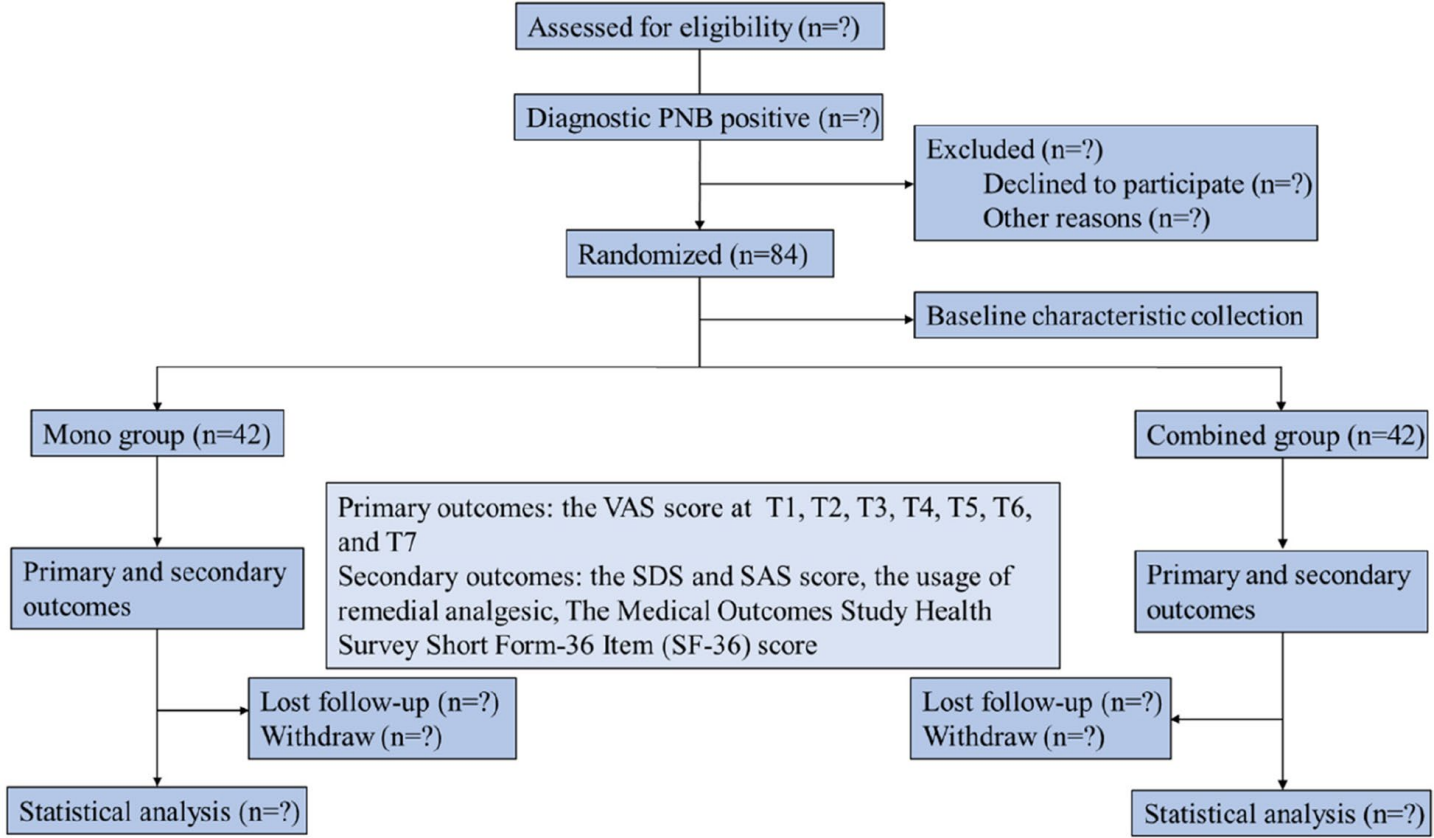
Consolidated Standards of Reporting Trial diagram for this trial. VAS, visual analog score; SAS, Self-Rating Anxiety Scale; SDS, Self-Rating Depression Scale; SF-36, Medical Outcomes Study Health Survey Short Form-36 Item (SF-36)

This is a multicenter prospective randomized, controlled trial with 1:1 allocation (Fig. 1). It has been registered at www.ChiCTR.org (ChiCTR2200061800). Ethical approval was granted by the Medical Ethics Committee of West China Hospital, Sichuan University. Written informed consent will be obtained from patients or their legal representatives by the designated attending physicians after explaining the trial to patients.

### Patients

The inclusion criteria for the study are as follows: (1) patients present with four essential clinical criteria for the diagnosis of PN based on the following Nantes criteria: (a) pain in the territory of the pudendal nerve (from the anus to the penis or clitoris); (b) pain is predominantly experienced while sitting; (c) pain does not typically disrupt sleep at night; (d) no objective sensory deficiency; (2) receiving analgesic medication for at least 2 weeks, but still has a VAS ≥ 4; (3) a VAS score decrease by more than 50% of the baseline after diagnostic PNB; (4) age 18–85 years.

The exclusion criteria for the study are as follows: (1) involved in other clinical trials within the last 3 months; (2) pregnant or plan to become pregnant; (3) heart disease; (4) nerve injury/central nervous system injury; (5) contraindications to injection with lidocaine, ropivacaine, mecobalamin, diprospan, or iodinated contrast medium; (6) active or recurrent urethral infection (more than five times in the last 12 months); (7) coagulation disorder or receiving anticoagulants; (8) received nerve block or PRF previously; and (9) refusal to participate in the trial.

### Randomization, concealment, and blinding

Randomly assigned sequences will be generated using SPSS 22.0 (IBM SPSS STATISTICS 22.0) by an independent research assistant, and the results will be sealed in an opaque envelope based on the chief investigator’s instructions. Eligible participants will be randomly assigned to the mono or combined groups at a 1:1 ratio. The allocation will not be revealed until all end point events have been evaluated for the last patient, or severe adverse effects have occurred. The patients and the attending physicians who conduct the treatments will not be blinded to the assigned treatment; however, the outcome assessors and statisticians will be blinded to the group assignments.

### Intervention

The designated attending physicians will conduct the PNB after admission, and evaluate the treatment effect 2 h after PNB. If the diagnostic PNB is positive (VAS score decreases by 50%), the patients will receive an allocation based on a random sequence by the chief investigator. The pudendal nerve PRF and GIB will also be conducted by the designated attending physicians at each center.

**Fig. 2:**
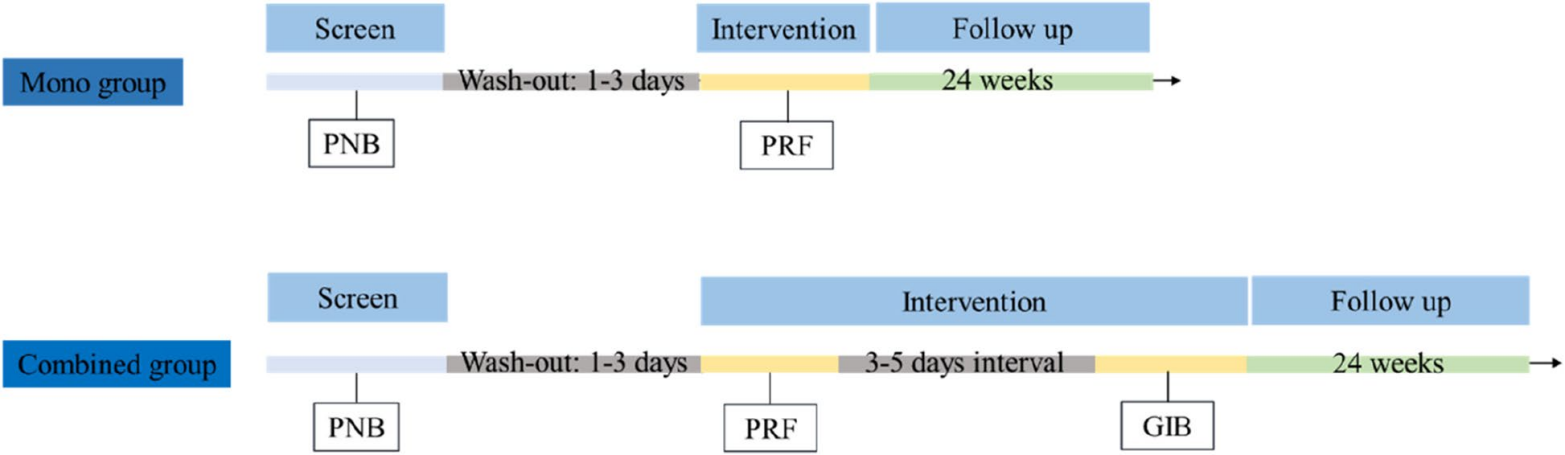
The study treatment schedule

The combined group will receive pudendal nerve PRF and GIB. The interval between PNB and PRF will be 1 day to 3 days, and that between PRF and GIB will be 3 days to 5 days (Fig. 2). The mono group will only receive pudendal nerve PRF. Permissible interventions during the RCT will include relevant concomitant care, such as pelvic floor physical treatment, pelvic floor magnetic stimulation, and transcranial magnetic stimulation. If the pain improvement is less than 30% or the VAS score is greater than four, remedial analgesia will be administered, such as pregabalin, oxycodone, and acetaminophen tablets.

All patients will be evaluated in person one day after the completion of therapy. Furthermore, patients will use an electronic device to answer a questionnaire on VAS, SAS, SDS, and SF-36 at the time of each visit. The follow-up evaluation in person or online will be conducted by outcome assessors, who will be blinded to allocation. The scores and data collection date will be recorded on the electronic device.

### PNB

**Fig. 3:**
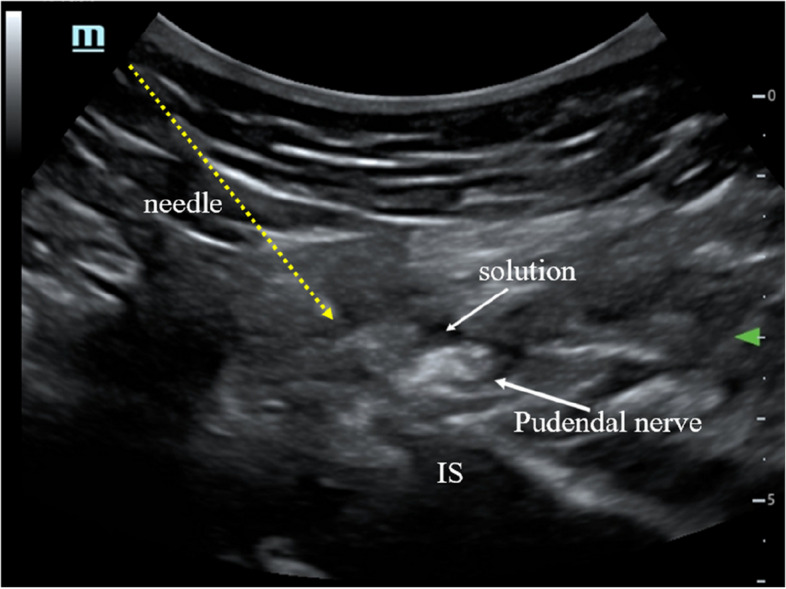
Pudendal nerve block or PRF treatment. IS, ischial spine

The patients will be in a prone position. First, an ultrasound transducer will be positioned at a transverse plane to visualize the ischium forming the lateral border of the sciatic notch. When the probe is moved in the cephalad–caudal direction, the length of the ischium gradually increases, with the widest point at the level of the ischial spine. The ischial spine will be identified by visualizing the internal pudendal artery again. Thereafter, a local anesthesia compound (2% lidocaine 1.5 ml + 1% ropivacaine 1.5 ml + 0.9% NaCl 2 ml) will be administered to the medial aspect of the internal pudendal artery (Fig. 3).

### Pudendal nerve PRF

The patients will be placed in a prone position. Under ultrasound guidance, the puncture needle will be inserted near the pudendal nerve (Fig. 3). After connecting the electrode and PRF needle, sensory stimulation at a frequency of 50 Hz, pulse width of 1 ms, and voltage of 0.3–0.5 V will be administered to produce paresthesia in the innervation of the pudendal nerve. PRF will be administered at 42℃ for 480 s. Thereafter, a 5-ml nerve nutrition compound (2 ml of 2% lidocaine + 1 ml of 0.5 mg mecobalamin + 1 ml diprospan + 1 ml of 0.9% NaCl) will be injected.

### GIB

**Fig. 4:**
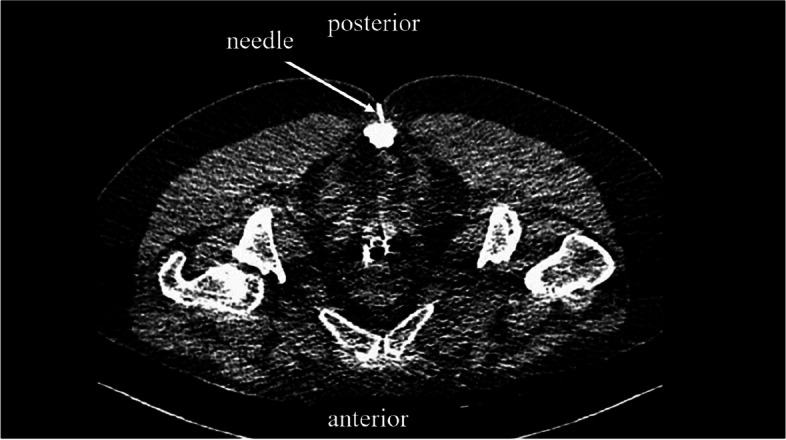
CT-guided GIB

The patients will be placed in a prone position, and the sacrococcygeal junction will be identified using the CT-guided frontal and lateral views. When the needle pierces through the sacrococcygeal ligament under CT guidance (Fig. 4), a loss of resistance will occur. The position of the needle tip will be confirmed by injecting a contrast medium. Subsequently, a local anesthesia compound (1.5 ml of 2% lidocaine + 1.5 ml of 1% ropivacaine + 1 ml diprospan + 1 ml of 0.9% NaCl) will be administered to block the ganglion impar.

### Outcomes

**Fig. 5:**
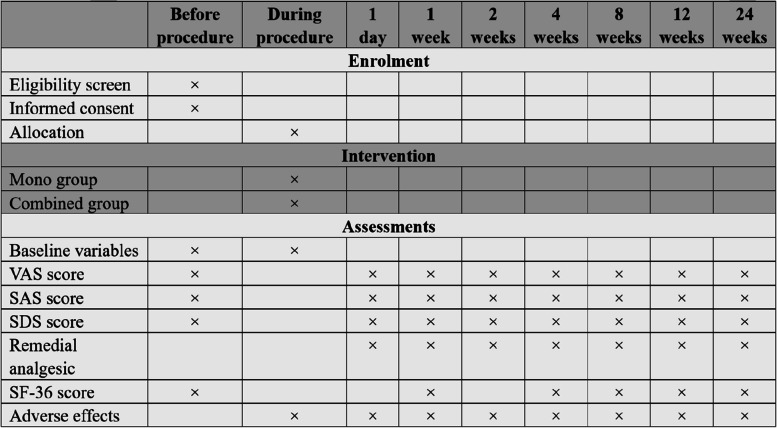
Schedule of enrollment, intervention, and assessments. VAS, Visual Analog Scale; SAS, Self-Rating Anxiety Scale; SDS, Self-Rating Depression Scale; SF-36, Medical Outcomes Study Health Survey Short Form-36 Item (SF-36)

The patient baseline characteristics will be documented at enrollment. The primary and secondary outcomes will be measured at 1 day (T1), 1 week (T2), 2 weeks (T3), 4 weeks (T4), 8 weeks (T5), 12 weeks (T6), and 24 weeks (T7) after the completion of treatment (Fig. 5). The collection of post-discharge outcomes will be performed by telephone interview or face-to-face when patients visit the clinic.

### Baseline characteristics

The following characteristics will be collected, including demographic characteristics (e.g., initial ailment, sex, age, parity, and history of vaginal delivery), medical history, duration of pain, and baseline score of VAS, SAS, SDS, and SF-36 scores.

### Primary outcome

The primary outcome will be the VAS score at T1, T2, T3, T4, T5, T6, and T7. The VAS score is a scale consisting of a line, usually 100 mm in length, from “no pain” (score of 0) to “worst imaginable pain” (score of 100).

### Secondary outcomes

The secondary outcomes include the following:

1. Self-Rating Anxiety Scale (SAS) and Self-Rating Depression Scale (SDS) scores at T1, T2, T3, T4, T5, T6, and T7. SAS and SDS developed by Zung will be used to screen for anxious and depressive disorders. The SAS and SDS contain 20 items that cover various anxiety and depression symptoms. Responses are provided on a 4-point scale from 1 to 4 [18, 19];
2. Use of remedial analgesic at T1, T2, T3, T4, T5, T6, and T7
3. The Medical Outcomes Study Health Survey Short Form-36 Item (SF-36) score at T2, T4, T5, T6, and T7. The SF-36 is widely used to assess quality of life in the Medical Outcomes Study. It contains eight domains: physical function; role physical; bodily pain; general health; vitality; social functioning; role emotional; and mental health. Higher scores indicate better health;
4. The incidence of adverse effects (AEs) (after/during/peri-procedural), including hypertension, hypotension, dizziness, nausea, vomiting, numbness of the legs, urinary retention, hematoma, and pain at puncture sites.

### Statistical analysis

Statistical analysis will be performed using SPSS 22.0 (IBM SPSS STATISTICS 22.0) according to the intention-to-treat and per-protocol analyses. The per-protocol set is defined as all patients who have undergone randomization and have received all treatment, except for those who dropped out because of poor efficacy. The Kolmogorov–Smirnov test will be used to evaluate the normality of the data distribution, and Levene’s test will be used to determine the homogeneity of variance. Continuous data will be represented as the mean with SD or median with the interquartile range based on its distribution. The categorical variable will be represented by counts and percentages. The difference between groups will be analyzed by a one-way analysis of variance, Mann–Whitney *U* test, chi-square, or Fisher’s exact tests as appropriate based on the type of variable. Generalized estimating equation analysis will be used for repeated measurement data. Missing data (e.g., loss to follow-up, death, withdrawal) will be updated using the multiple-imputation approach. A two-sided *P* value of 0.05 will be considered statistically significant.

### Sample size

According to a previous study, the VAS score was 3.6 ± 1.9 after 1 month of PRF [10]. We assumed that the pudendal nerve PRF with GIB will decrease the VAS score by 30% with a total two-sided type I error rate of 0.05 and a 20% withdrawal rate. A total of 84 patients will be enrolled to achieve a statistical power of 80%.

### Data collection and management

#### Data collection

Before recruitment, all investigators will be invited to evaluate the design and explore the feasibility of the intervention. Standard operating procedures will be established in detail. All investigators will be trained on the grading rules of scales and matters that need attention to guarantee the quality of the trial. The procedures will be carried out by designated pain physicians and follow-up will be performed by a trained research assistant.

#### Data management

Data will be recorded in a case report form. The research documents will be locked in a single secure office, and the key will be kept by the chief investigator until the end of the study.

### Oversight and monitoring

#### Composition of the data monitoring committee, its role, and reporting structure

The chief investigator and other investigators are doctors and professors who will be responsible for overseeing and monitoring the entire study. The periodical meetings will include the following: (1) study investigators report the process of the study, including recruitment, informed consent, assessment, and intervention procedures according to the protocol; (2) study investigators raise problems during the performance of the study; and (3) chief investigators provide oversight for all aspects of the study and support to the study investigators. The study team will meet bimonthly with the data testing committee to monitor the progress and quality of the study.

#### Interim analyses

No interim analyses will be planned because of the low-risk interventions, short-term duration, and small sample size. If a patient reports serious AEs or deterioration during the study, the chief investigator may decide to terminate the trial.

#### Reporting and treatment of AEs

The investigators will monitor and record the AEs immediately after the event and conduct follow-up until the adverse events are resolved. If the AEs are common and the symptoms are mild, we will supply treatments with close monitoring. If the AEs become severe, the intervention will be stopped and unblinded as necessary. Professional doctors from various fields will immediately assist the patients. All AEs will be documented and reported to the institutional review board by the chief investigator. The chief investigator conducts regular a cumulative analysis of all AEs, and investigator meetings will be conducted as necessary to evaluate risks and benefits.

Treatment of AEs during the procedure:

1. If the heart rate is below 50 bpm, a bolus of 0.25 mg atropine will be administered until the heart rate is above 60 bpm.
2. If the systolic blood pressure decreases by more than 20% of the baseline, a bolus of 0.2 mg metaraminol will be administered. Fluid will also be administered as necessary.
3. If symptoms, such as dizziness, a metallic taste in the mouth, and vomiting manifest, a local anesthetic intoxication-related procedure will be carried out.

Treatment of AEs after the procedure.

1. If the patient complains about nausea and vomiting, a 5-HT receptor antagonist will be administered.
2. If the patient complains about pruritus, the intervention will be stopped.
3. If the patient complains about numbness of the lower limb, or lower limb dyskinesia, intervention will be immediately interrupted. Neurosurgery consultation is warranted if symptoms do not improve.

## Patient and public involvement

Patients or the public will be not involved in the design, implementation, reporting, or dissemination plans of the study.

## Ethics and dissemination

This study will be conducted in accordance with the ethical principles stipulated in the *Declaration of Helsinki* and *Ethical Review of Human Biomedical Research* established by the National Health Commission of the People’s Republic of China. All patients will receive verbal and written information and must provide written informed consent before enrolment. The study will be conducted after the institutional review board’s approval, and any amendments to the protocol will be submitted to the institutional review board. We will publish our findings in peer-reviewed journals.

## Discussion

A prospective, randomized, controlled study will be conducted to evaluate the efficacy and safety of pudendal nerve PRF with GIB in PN.

Ultrasound-guided pudendal nerve PRF and CT-guided GIB have been widely used to treat chronic pain. To date, some retrospective studies, case reports, and case series on the efficacy of PRF or GIB in PN have been reported [13–15, 17, 20–22]. However, the ultrasound-guided PRF of the pudendal nerve combined with GIB is used in PN, but randomized controlled trials are lacking. In the above studies, the PRF parameters were set as follows: temperature ranging from 42 ℃ to 45 ℃ with a duration from 120 to 900 s. Although the high-voltage long-duration PRF mode shows better improvement in PN [12], based on our clinical experience and to avoid discomfort, PRF will be administered at 42 ℃ for 480 s in our study. Our primary outcome will be the efficacy of PRF combined with GIB for the long-term improvement of PN. At long-term follow-up (ranging from 2.3 years to 8.8 years), Krijnen et al. [13] found that most patients received repeated PRF therapy every 2 to 6 months. Thus, the follow-up will last for 24 weeks in our study.

Nevertheless, our study will have several limitations. First, the memory effect is an important mechanism in the development of chronic pain, and nerve block or PRF will be repeatedly implemented with an interval from a few days to weeks [23]. However, the patients in this study will receive only one pudendal nerve PR. Second, the success of pudendal PRF may be affected by the anatomical level of nerve injury. Interligamentous pudendal nerve entrapment patients experience more benefits than those having pudendal nerve entrapment in the endopelvic portion [8].

Overall, our study results will provide valuable information on the potential benefits of this combination therapy and contribute to the development of more effective and safe treatments for PN patients.

## Trial status

Recruiting patients is ongoing at the time of manuscript submission. Recruitment began on 1 May 2023 and is expected to be complete by December 2023.

Protocol version 3.0 (20230321).

## Data Availability

Not applicable. No data have been generated.

## Abbreviations

PN: Pudendal neuralgia
PNB: Pudendal nerve block
CRF: Continuous radiofrequency
PRF: Pulsed radiofrequency
GIB: Ganglion impar block
VAS: Visual analog score
SAS: Self-Rating Anxiety Acale
SDS: Self-Rating Depression Scale
AE: Adverse effect
